# Supplementary material for: Point of Care Lung Ultrasound Injury Score—A simple and reliable assessment tool in COVID-19 patients (PLIS I): A retrospective study
Source: PLoS One. 2022 May 11;17(5):e0267506. doi: 10.1371/journal.pone.0267506 (PMC9094523; doi:10.1371/journal.pone.0267506)
Supplement: Attachment — Submitted filename: PONE- answering letter .docx [file pone.0267506.s001.docx]

Answer letter to:

PONE-D-21-38198

Point of Care Lung Ultrasound Injury Score - a Simple and Reliable Assessment Tool in COVID-19 Patients (PLIS I): A Retrospective Study

PLOS ONE

Dear Editor,

Thank to you and to the reviewers, we believe that the manuscript now is better, more clear to the readers, and improved:

1. Please ensure that your manuscript meets PLOS ONE's style requirements, including those for file naming. The PLOS ONE style templates can be found at
   <https://journals.plos.org/plosone/s/file?id=wjVg/PLOSOne_formatting_sample_main_body.pdf> and 
   <https://journals.plos.org/plosone/s/file?id=ba62/PLOSOne_formatting_sample_title_authors_affiliations.pdf>

Answer: **Done**

2. answer: “**The authors received no specific funding for this work.”**

Please include your amended statements within your cover letter; we will change the online submission form on your behalf.

Answer: **Done**

3. Thank you for stating the following in the Competing Interests section: "Lior Fuchs is a consultant for GE Healthcare."

Please confirm that this does not alter your adherence to all PLOS ONE policies on sharing data and materials, by including the following statement: "This does not alter our adherence to  PLOS ONE policies on sharing data and materials.” (as detailed online in our guide for authors <http://journals.plos.org/plosone/s/competing-interests>).  If there are restrictions on sharing of data and/or materials, please state these. Please note that we cannot proceed with consideration of your article until this information has been declared.

Please include your updated Competing Interests statement in your cover letter; we will change the online submission form on your behalf.

Answer: **I – Lior Fuchs, declare that: "This does not alter our adherence to PLOS ONE policies on sharing data and materials.”**

**It is updated in the cover letter.**

4. In your Data Availability statement, you have not specified where the minimal data set underlying the results described in your manuscript can be found. PLOS defines a study's minimal data set as the underlying data used to reach the conclusions drawn in the manuscript and any additional data required to replicate the reported study findings in their entirety. All PLOS journals require that the minimal data set be made fully available. For more information about our data policy, please see <http://journals.plos.org/plosone/s/data-availability>.

Upon re-submitting your revised manuscript, please upload your study’s minimal underlying data set as either Supporting Information files or to a stable, public repository and include the relevant URLs, DOIs, or accession numbers within your revised cover letter. For a list of acceptable repositories, please see [http://journals.plos.org/plosone/s/data-availability#loc-recommended-repositories](http://journals.plos.org/plosone/s/data-availability#loc-recommended-repositories). Any potentially identifying patient information must be fully anonymized.

Important: If there are ethical or legal restrictions to sharing your data publicly, please explain these restrictions in detail. Please see our guidelines for more information on what we consider unacceptable restrictions to publicly sharing data: [http://journals.plos.org/plosone/s/data-availability#loc-unacceptable-data-access-restrictions](http://journals.plos.org/plosone/s/data-availability#loc-unacceptable-data-access-restrictions). Note that it is not acceptable for the authors to be the sole named individuals responsible for ensuring data access.

We will update your Data Availability statement to reflect the information you provide in your cover letter.

**Answer:** unfortunately, there are ethical and legal restrictions to sharing our data publicly:

Based on Clalit Health Services regulations, the study data set maintain only at Clalit Health Services servers. Researchers are not allowed to upload or share the data set with other sources.

5. Please amend the manuscript submission data (via Edit Submission) to include author Khaled el haj, MD:

**Answer:** Done

6. Your ethics statement should only appear in the Methods section of your manuscript. If your ethics statement is written in any section besides the Methods, please delete it from any other section.

**Answer:** Done

7. Please upload a new copy of Figures 2 and 3 as the detail is not clear. Please follow the link for more information: <https://blogs.plos.org/plos/2019/06/looking-good-tips-for-creating-your-plos-figures-graphics/>" <https://blogs.plos.org/plos/2019/06/looking-good-tips-for-creating-your-plos-figures-graphics/>

**Answer:** Done

8. Please review your reference list to ensure that it is complete and correct. If you have cited papers that have been retracted, please include the rationale for doing so in the manuscript text, or remove these references and replace them with relevant current references. Any changes to the reference list should be mentioned in the rebuttal letter that accompanies your revised manuscript. If you need to cite a retracted article, indicate the article’s retracted status in the References list and also include a citation and full reference for the retraction notice.

**Answer:** Sorry for the inconsistency with the references. There was a technical problem with the order of the references. I have reviewed the reference list and corrected it. No references were retracted. All original references are labeled by the PLOS one font instructions.

Reviewers' comments:

Reviewer's Responses to Questions

**Comments to the Author**

1. Is the manuscript technically sound, and do the data support the conclusions?

The manuscript must describe a technically sound piece of scientific research with data that supports the conclusions. Experiments must have been conducted rigorously, with appropriate controls, replication, and sample sizes. The conclusions must be drawn appropriately based on the data presented.

Reviewer #1: Yes

Reviewer #2: Yes

2. Has the statistical analysis been performed appropriately and rigorously?

Reviewer #1: Yes

Reviewer #2: Yes

3. Have the authors made all data underlying the findings in their manuscript fully available?

The [PLOS Data policy](http://www.plosone.org/static/policies.action#sharing) requires authors to make all data underlying the findings described in their manuscript fully available without restriction, with rare exception (please refer to the Data Availability Statement in the manuscript PDF file). The data should be provided as part of the manuscript or its supporting information, or deposited to a public repository. For example, in addition to summary statistics, the data points behind means, medians and variance measures should be available. If there are restrictions on publicly sharing data—e.g. participant privacy or use of data from a third party—those must be specified.

Reviewer #1: Yes

Reviewer #2: Yes

4. Is the manuscript presented in an intelligible fashion and written in standard English?

PLOS ONE does not copyedit accepted manuscripts, so the language in submitted articles must be clear, correct, and unambiguous. Any typographical or grammatical errors should be corrected at revision, so please note any specific errors here.

Reviewer #1: Yes

Reviewer #2: Yes

5. Review Comments to the Author

Please use the space provided to explain your answers to the questions above. You may also include additional comments for the author, including concerns about dual publication, research ethics, or publication ethics. (Please upload your review as an attachment if it exceeds 20,000 characters)

Reviewer #1: Thanks for this interesting paper on the use of ultrasound in COVID patients.

My comments are :

Background : could you provide some references to “There is a direct association between the severity of COVID-19 pneumonia and computerized tomography (CT) findings.”

**Answer:** there were errors with the references labels – now we provide the right references at the right place, as well as the one in your comment.

Could you add something about the predominant COVID variant in your hospital during the study?

**Answer:**  The study period included the Alpha, and Beta SARS-CoV-2 variants.

We added this sentence to the first paragraph of the Methods.

Could you please address this comment: This is a retrospective study, although data were prospectively collected.
Was the study designed prospectively?? Was it registered? Could you discuss how the study was designed?

**Answer:** we agree that the term “prospectively collected “ is confusing. This is a retrospective study that analyses retrospectively a new clinical lung US score that was implemented during the COVID 19 outbreak. We removed the term prospectively collected and added it to the methods section:

“This is a retrospective study; data were collected during patients assessments and rounds. The PLIS protocol was taught and implemented in the COVID 19 wards and ICUs and was part of the daily patients assessment.”

Later in Methods, we further explain the way we collected the data: “Data collected included the PLIS recorded on admission, when conducted during ward/ICU rounds, and when otherwise clinically indicated.”

This was a routine lung assessment for all. All about it is a retrospective analysis.

An important question is the timing of the data collection. The COVID time course is useful in understanding deterioration, and I am not sure from the manuscript whether the measures on patients reflect – paired measurements (ED or ward then ICU, or in some patients were single measurements, or in others, multiple measurements on the same patient). It just states “on admission” – not sure whether that is to hospital or the ward or to ICU.

**Answer:**  Thank you for the important comment. For clarification: this is not a prospective study, and PLIS exams were not protocolized. Some of the patients received only ward PLIS assessments as they were never admitted to the ICU. Some received only in the ICU and not in the ward. Some were received in both locations. The studies were not paired.

We added the following sentence to the second paragraph in the Methods:

“*As this is not a prospective study, the PLIS exams were not protocolized. Some patients could receive a PLIS study on regular ward admission, during the morning rounds, and also later, when admitted to the ICU. Some received ultrasound lung scan only during the ICU admission or only in the regular ward.”*

P 5 “were assessed by an intensivist to require mechanical ventilation in the next 24 hours potentially. “
Were assessed by an intensivist to potentially require …

**Answer:** Thanks, we have added “potentially”.

Figure 1: why are the stars in a different position to the probe?

**Answer:**The stars represent the area of scan in each zone and not the exact place of the probe. This comment was added to the Figure 1 legend.

For the “putting it together,” could you create a figure that shows perhaps the different components with the ultrasound images ?

**Answer:** Thank you for an excellent idea. **Figures 1b** and **1c** with b lines and consolidation were added and referred to in the putting it together paragraph.

Interobserver variability – how many scans were repeated by different operators?

**Answer:** Inter-observer reliability was assessed by comparing independent ratings of the PLIS B and C scores among 16 randomly selected patients in a blinded manner by two physicians. Please see the Statistical Analysis section for more details.

Table 4 Did you run the model just with PLIS A? Just wanting to understand the added benefit of the U/S scan in predictions.

**Answer:** PLIS A is connected to the prognosis, as we know from previous data regarding the outcomes of SARS- 2 ventilated patients. This is the reason we present Figure 2, which shows the association between the SOFA, PLIS B and PLIS C. We also analyzed the association between these lung ultrasound scores (PLIS B and C, independently from PLIS A) and other outcomes, as the risk for mechanical ventilation. We found OR of over 4 and over 10 for PLIS B2 and PLIS C2, respectively, compared to PLIS B0 and C0. So, we do know that the ultrasound score is a predictor and associated with bad outcomes, regardless of the PLIS A score. We chose to publish these specific associations in PLIS II report.

Reviewer #2: This is an interesting paper which validates some of our regular utility of sonography in the ICU during the pandemic. We have already appreciated that CT scan findings with higher "scores" i.e. worse diseases, likely have a poorer outcomes. This essentially is also shown in this paper where the authors evaluate the lung parenchyma.

Using PLIS in their group is sound however this group may not reflect the prevalence of underlying diseases in other countries and so this should be taken with a grain of salt. For example, CKD or CHF may be more prevalent in certain populations which would affect the lung sonographic profile.

**Answer:** I strongly concur. We have added this sentence to the Limitations paragraph.

6. PLOS authors have the option to publish the peer review history of their article ([what does this mean?](https://journals.plos.org/plosone/s/editorial-and-peer-review-process#loc-peer-review-history)). If published, this will include your full peer review and any attached files.


If you choose “no”, your identity will remain anonymous but your review may still be made public.


**Do you want your identity to be public for this peer review?** For information about this choice, including consent withdrawal, please see our [Privacy Policy](https://www.plos.org/privacy-policy).

Reviewer #1: No

Reviewer #2: **Yes:** Kinner Patel

[NOTE: If reviewer comments were submitted as an attachment file, they will be attached to this email and accessible via the submission site. Please log into your account, locate the manuscript record, and check for the action link "View Attachments". If this link does not appear, there are no attachment files.]

While revising your submission, please upload your figure files to the Preflight Analysis and Conversion Engine (PACE) digital diagnostic tool, <https://pacev2.apexcovantage.com/>. PACE helps ensure that figures meet PLOS requirements. To use PACE, you must first register as a user. Registration is free. Then, login and navigate to the UPLOAD tab, where you will find detailed instructions on how to use the tool. If you encounter any issues or have any questions when using PACE, please email PLOS at [figures@plos.org](mailto:figures@plos.org). Please note that Supporting Information files do not need this step.
